# Consensus from European experts on severe eosinophilic asthma and chronic rhinosinusitis with nasal polyps: Results from the OverSEA Delphi study

**DOI:** 10.1016/j.jacig.2025.100529

**Published:** 2025-07-03

**Authors:** Claus Bachert, Guy Brusselle, José Antonio Castillo Vizuete, Ignacio Dávila, Martin Laudien, Veronica Seccia, Peter Schmid-Grendelmeier, Alessandra Vultaggio, Konstantina Kallinikou, Laura Walrave, Ludger Klimek

**Affiliations:** a Department of Otorhinolaryngology, Head and Neck Surgery and the German Center of Diseases of the Upper Airway, University Hospital Münster, Münster, and the Department of Otorhinolaryngology, First Affiliated Hospital of Sun Yat-sen University, International Airway Research Center, Guangzhou, China; b Department of Respiratory Medicine, Ghent University Hospital, Ghent, Belgium; c Department of Pulmonology, Hospital Universitari Dexeus, and the Group of Rhinitis, Rhinosinusitis and Nasal Polyps, Area of Asthma, SEPAR, Barcelona, Spain; d Allergy Service, University Hospital of Salamanca, Biomedical and Diagnosis Science Department, Salamanca University School of Medicine, Salamanca, Spain; e Department of Otorhinolaryngology, Head and Neck Surgery, Kiel University, University Medical Centre Schleswig-Holstein, Kiel, Germany; f Otolaryngology Audiology and Phoniatric Operative Unit, Department of Surgical, Medical, Molecular Pathology and Critical Care Medicine, Azienda Ospedaliero Universitaria Pisana, University of Pisa, Pisa, Italy; g Allergy Unit, Department of Dermatology, University Hospital Zurich, Zurich, Switzerland; h Immunoallergology Unit, Careggi University Hospital, Department of Experimental and Clinical Medicine, University of Florence, Florence, Italy; i GSK, Medical Affairs, Athens, Greece; j GSK, Medical Affairs, Wavre, Belgium; k Center for Rhinology and Allergy, Wiesbaden, Germany

**Keywords:** Severe asthma, type 2 inflammation, eosinophilic phenotype, chronic rhinosinusitis, nasal polyps, comorbidities, biomarkers, biologics, multidisciplinary

## Abstract

**Background:**

Managing patients with severe asthma with an eosinophilic phenotype (SEA) with comorbid respiratory conditions such as chronic rhinosinusitis with nasal polyps (CRSwNP) continues to encounter significant challenges and lack of coordinated management among treating physicians.

**Objective:**

The OverSEA study aims to provide insights into current clinical practices and formulate recommendations for managing these patients.

**Methods:**

The two-round Delphi survey, conducted March-June 2023, was developed by a multidisciplinary 11-member Scientific Committee including pulmonologists, allergists, and ear, nose and throat specialists, and involved 205 experts from these specialties across 8 European countries. Consensus was defined as ≥70% agreement. Topics covered included the initial assessment, treatment, follow-up, and multidisciplinary management of patients with SEA and CRSwNP.

**Results:**

There was a consensus that evaluating for CRSwNP (88%), allergic rhinitis (79%), chronic rhinosinusitis without nasal polyps (77%), and aspirin/nonsteroidal anti-inflammatory-exacerbated respiratory disease (71%) is crucial for diagnosing upper respiratory tract comorbidities in patients with SEA. The necessity of a multidisciplinary approach for all stages of disease management (diagnosis, 82%; treatment decision-making, 83%, follow-up, 79%), and the usefulness of biologics in simultaneously managing asthma and CRSwNP symptoms (87%) were emphasized.

**Conclusion:**

The OverSEA study is the largest European initiative providing recommendations for optimizing the management of patients with SEA and comorbid CRSwNP. It underscores the importance of evaluating patients with SEA for comorbid upper airways diseases, particularly CRSwNP, and promotes a multidisciplinary approach, encouraging pulmonologists, allergists, and otorhinolaryngologists to collaborate closely to streamline patient diagnosis, follow-up, and treatment decisions.

Severe asthma with an eosinophilic phenotype (SEA) is a chronic respiratory disease that imposes a significant burden on patients and health care systems.^1^, ^2^, ^3^, ^4^, ^5^ Chronic rhinosinusitis with nasal polyps (CRSwNP) is a common comorbidity in patients with SEA, affecting 57-62% of patients with severe asthma and 38-42% of those with mild to moderate asthma.^6^ In patients with SEA, the burden of asthma is worsened by upper respiratory airway disease, leading to additional challenges such as sleep disturbances, fatigue, depression, and a decrease in overall well-being.^7^, ^8^, ^9^ Pathologically, severe asthma and CRSwNP share similar underlying mechanisms, featuring type 2 inflammation and raised eosinophil levels in the lower and upper airways.^10^, ^11^, ^12^

The therapeutic management of patients with severe asthma and CRSwNP typically includes inhaled corticosteroids, other controller medications such as long-acting bronchodilators, nasal irrigation, intranasal corticosteroid therapy, or systemic corticosteroid (SCS) therapy.^7^, ^8^, ^9^ However, a significant number of patients with SEA and CRSwNP either become unresponsive to these treatments, experience exacerbations, require secondary/revision endoscopic sinus surgery, or require increased/continuous receipt of SCS therapy.^7^^,^^9^

Patients with SEA and CRSwNP may be eligible for biologic therapy, which targets specific underlying type 2 inflammatory mediators,^13^ after certain therapeutic interventions have been exhausted.^7^, ^8^, ^9^ Currently, 6 monoclonal antibody therapies (aka biologics) are approved for the treatment of severe asthma: omalizumab,^14^ mepolizumab,^15^ reslizumab,^16^ benralizumab,^17^ dupilumab,^18^ and tezepelumab;^19^ and 3 monoclonal antibody therapies are approved for severe uncontrolled CRSwNP: omalizumab,^14^ mepolizumab,^15^ and dupilumab.^18^ However, there are significant challenges in the treatment pathway for patients with SEA with CRSwNP, including delays in diagnosing upper respiratory conditions including CRSwNP, heterogeneity and disparities in evaluating treatment progress and making treatment decisions, and absence of specific guidelines for comorbid patients for use by multidisciplinary teams for the initial assessment, treatment, and follow-up of patients with SEA and CRSwNP. Although guidelines include specific recommendations on the management of patients with comorbid SEA and CRSwNP,^13^ they may not always be implemented into clinical practice. An expert consensus study was thus chosen to systematically and pragmatically capture the disease management strategies that are used by specialists in clinical practice across several European countries. The Delphi technique is a recognized group facilitation technique that is used to gain consensus via collation of data through expert respondents.

The overarching objective of the OverSEA project was to achieve consensus on recommendations from European experts for the initial assessment, diagnosis, treatment, referral, and follow-up of patients with SEA with comorbidities, particularly CRSwNP. These recommendations aim to guide respiratory physicians, including pulmonologists, allergists, and otorhinolaryngologists, in the effective multidisciplinary management of patients with SEA and comorbid CRSwNP.

## Methods

### Selection of panelists

The full methods used to develop the OverSEA Delphi consensus are described in Fig E1 and the themes and statements in Table E1, both in this article’s Online Repository available at www.jaci-global.org. A European Scientific Committee composed of 11 experts including pulmonologists (G.B. and J.A.C.V., and Wolfgang Pohl), allergists (I.D., P.S.G., and A.V.), and otorhinolaryngologists (C.B., M.L., V.S., L.K., and Jean-François Papon) developed a two-round Delphi survey that involved a multidisciplinary panel of 205 European experts. This panel, with members hailing from 8 European countries (Austria, Belgium, France, Germany, Italy, Spain, Switzerland, and the United Kingdom), included 156 pulmonologists and 28 allergists, all with significant experience in managing patients with severe asthma and related comorbidities, particularly CRSwNP. Additionally, 21 otorhinolaryngologists were part of the panel, contributing their expertise in multidisciplinary collaboration with pulmonologists. The panel was selected on the basis of the requirement to include a balanced number of specialists from each participating European country. The selection of participating countries was based on a balance between larger and smaller European nations. An invitation email that complied with the European General Data Protection Regulation containing study information and objectives was sent to panel members who met the criteria for participation; participation was entirely voluntary and anonymous. Each round took approximately 5 weeks to complete; the first round took place in March and April 2023, followed by the second round in May and June 2023.

### Preparatory research

Two Delphi questionnaires were developed by the Scientific Committee on the basis of literature identified as key, and relevant clinical practice guidelines. Although a systematic literature search was not conducted, an *ad hoc* narrative PubMed search was performed without the use of any specific search strings. The purpose of this *ad hoc* narrative search was to identify additional articles and guidelines that were related to patient-reported outcomes used in CRSwNP in order to assess disease evolution. Relevant articles were used to generate the statements included in both questionnaires.

### Assessing consensus

To reach a consensus on a series of statements (Table E1), an iterative approach was developed following a series of qualitative and quantitative methods based on the Accurate Consensus Reporting Document guidelines.^20^ No formal protocol was registered for this study. Delphi methodology was used to achieve a European expert perspective on various aspects of SEA and CRSwNP.^21^, ^22^, ^23^, ^24^ The survey covered: (1) panelist profile and patients, (2) current management strategies, and (3) recommendations for patients with SEA and CRSwNP (initial assessment, treatment, follow-up, and multidisciplinary approach).

The Delphi questionnaire was completed online in two rounds; study materials were not piloted. In the first round, panel members individually rated their agreement with each statement on a 9-point Likert scale, as follows: 1-3, completely disagree; 4-6, neutral; and 7-9, completely agree. Consensus was defined by the Scientific Committee, who did not participate in the surveys, based on previous Delphi consensus studies, as ≥70% of panelists either completely agreeing or disagreeing with a statement. Anonymity was guaranteed by using numbers for identification of responses and not collecting any personally identifiable information.

After the first round, statements that did not reach consensus were revised and clarified by the Scientific Committee. The second round consisted of the revised statements that failed to achieve consensus in the first round (ie, statements agreed by ≥70% of panelists in the first round were excluded from the second round); this was conducted to secure a higher possibility of consensus that was not clearly determined in the first round. The responses were synthesized through quantitative analysis; descriptive statistics were used to assess the distribution of responses and identify areas of agreement or divergence. Where open-ended responses were sought, qualitative analysis was conducted, but no formal methods were applied. The final consensus level for all statements is presented.

### Participation

Panel members were reimbursed at local fair market value for their time spent answering the questionnaires. The questionnaires and online platforms were translated to local languages to ensure accessibility across the participating European countries.

## Results

### Response rate

The overall response rate was approximately 30.0%, with 234 and 205 participants responding to the first and second round of questionnaires, respectively, and the dropout rate (defined as the proportion of participants who participated in the first round but not the second) was 12.4%. No statements were removed or modified during the consensus process. There were no extensions or delays in the analysis phase.

### Panelist characteristics

Overall, 75% of the panelists worked in a public hospital, 16% worked in both public and private sectors, and 64% were affiliated with teaching hospitals. Most panel members had more than 10 years of professional experience, with 80% of panelists being members of either international or national scientific societies. Over the past 12 months, pulmonologists (n = 156) and allergists (n = 28) treated an average of 145 and 133 patients with SEA, respectively, with 31.4% and 40.9% of these patients also having comorbid CRSwNP. Otorhinolaryngologists (n = 21) had treated an average of 335 patients with CRSwNP over the past 12 months, with 34.9% of these patients presenting with comorbid SEA.

Overall, 26% of patients treated by a pulmonologist received oral corticosteroid (OCS) therapy and 35% received a biologic treatment, while 25% of patients treated by an allergist received OCS and 40% were treated with a biologic. For patients with CRSwNP, 44% had previous surgery for nasal polyps (NP), 33% received OCS, and 31% were treated with a biologic. In total, between 29% and 40% of patients with SEA initiated treatment with a biologic because of a diagnosis of NP with the goal of simultaneously treating both pathologies.

### Initial assessment of upper respiratory tract comorbidities

The expert panel was asked which upper respiratory tract comorbidities should optimally be assessed for in patients with SEA (Table E1). There was a consensus (≥70%) that an evaluation for CRSwNP (88%), allergic rhinitis (79%), chronic rhinosinusitis without NPs (77%), and aspirin/nonsteroidal anti-inflammatory-exacerbated respiratory disease (AERD/NERD, 71%) is essential for an accurate diagnosis of upper respiratory tract comorbidities in patients with SEA during their initial assessment. There was agreement that assessing upper respiratory tract comorbidities could be beneficial in reducing OCS and their potential adverse effects (90%), improving quality of life of patients (90%) and asthma control (87%), facilitating optimal therapeutic management (87%), and ensuring referral to the appropriate specialist (83%) for these patients (Table E1). The consensus results were consistent with those reported as routinely performed in the panel’s clinical practice, except for otorhinolaryngologists, who did not reach a consensus on the importance of assessing for the presence of AERD/NERD (48%).

**Fig 1:**
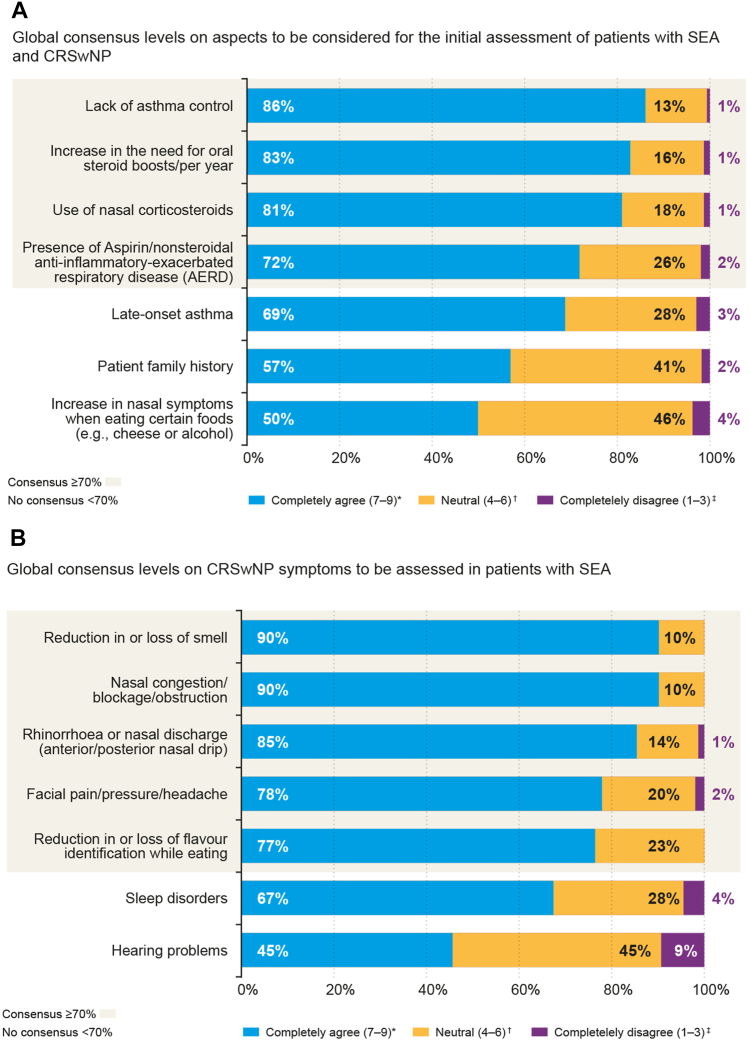
Level of global consensus (all specialties; pulmonologists, allergists, and otorhinolaryngologists) on aspects to be considered for initial assessment of patients with SEA with CRSwNP **(A)** and for CRSwNP symptoms to be assessed in patients with SEA **(B)**. ∗Completely agree was defined as Likert score of 7-9; †neutral, 4-6, and ‡completely disagree, 1-3.

Regarding upper respiratory tract comorbidities and CRSwNP, it was agreed that the assessment of CRSwNP should include the evaluation of NP-associated clinical symptoms, including a decrease or loss of smell (90%), nasal congestion (90%), rhinorrhea or nasal discharge (85%), facial pain, pressure, or headache (78%), and a decrease or loss of taste (77%) (Fig 1). In addition, agreed-on aspects to evaluate in comorbid patients with SEA and CRSwNP were lack of asthma control (85%), increase in the need for OCS boosts per year (77%), receipt of nasal corticosteroids (77%), and presence of AERD/NERD (77%) (Fig 1). No consensus was reached for the assessment of late-onset asthma (67%).

Focusing on the clinical tests to be performed during the initial assessment of CRSwNP in patients with SEA (Table E1), there was consensus on the use of nasal endoscopy (84%) and an NP grading system (74%). Pulmonologists and allergists also recommended complementing endoscopy with radiologic/imaging techniques (global consensus, 73%; pulmonologists, 71%; allergists, 93%), whereas otorhinolaryngologists did not reach consensus on the need for additional radiologic/imaging for CRSwNP (62%).

Regarding the use of biomarkers in the initial assessment of patients (Table E1), the panel agreed on the importance of blood eosinophil levels (88%) and fractional exhaled nitric oxide (Feno; 78%), although not on nasal tissue eosinophil levels (53%) or sputum eosinophil counts (53%). The specialty-wise analysis revealed that all groups agreed on the use of blood eosinophil levels, but only pulmonologists and allergists found Feno useful (pulmonologists, 78%; allergists, 86%; otorhinolaryngologists, 38%).

**Fig 2:**
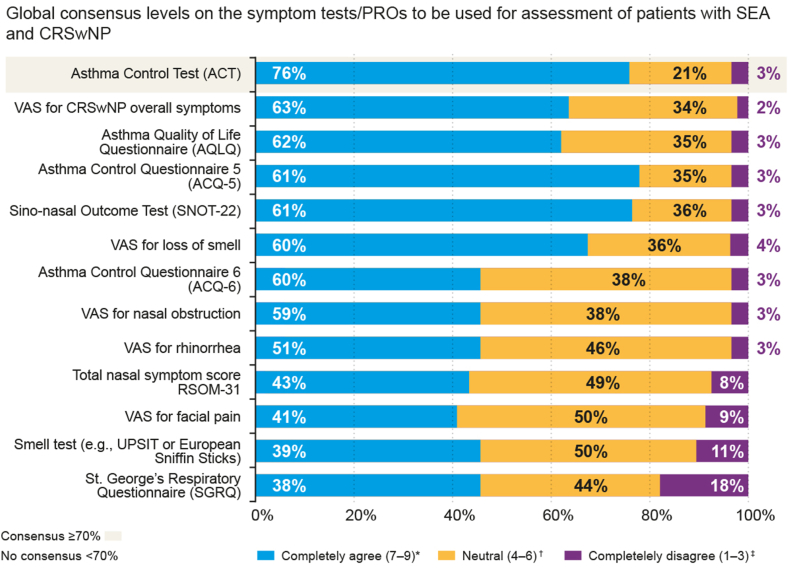
Level of global consensus (all specialties; pulmonologists, allergists, and otorhinolaryngologists) on symptom tests/PROs to be used for assessment of patients with SEA and CRSwNP. ∗Completely agree was defined as Likert score of 7-9; †neutral, 4-6, and ‡completely disagree, 1-3.

A similar lack of consensus was observed for the available symptom tests or patient-reported outcomes (PROs) that were presented to the panel (Table E1), with the Asthma Control Test (ACT) being the only one to achieve global consensus (76%) (Fig 2) and individual consensus among pulmonologists (78%) and allergists (86%). Despite the absence of consensus among otorhinolaryngologists on any symptom tests or PROs to be used for CRSwNP, it is worth noting that the Sinonasal Outcome Test 22 (SNOT-22) received their highest score (62%).

While panelists reached consensus on the importance of testing the reduced or lost sense of smell and evaluating patient quality of life (when expressed with general statements), no specific PRO or visual analog scale test emerged as a clear favorite (Fig 2).

### Treatment and follow-up

There was a global consensus that the primary treatment objectives should be to reduce asthma exacerbations and attain asthma control while simultaneously addressing CRSwNP symptoms and NP size. This should ideally be achieved through an OCS-sparing regimen, with the aim of minimizing the burden of adverse events and enhancing patients’ overall quality of life (>70% in all) (Table E1).

There was also a consensus that the treatment decision-making process and follow-up (Table E1) should be a collaborative effort between pulmonologists, allergists, and otorhinolaryngologists (83%). Toward this goal, there was consensus on the need for novel therapies to address unmet needs (81%) and the requirement to revise pharmacologic therapy in the event of disease progression (88%). However, the level of consensus varied for other aspects, with differences emerging that were based on specialty. Recurrent SCS therapy as an indicator of poor disease control was agreed on by 83% of panelists, but this was primarily supported by pulmonologists (87%) and otorhinolaryngologists (86%) and less so by allergists (64%). Similarly, there was agreement on reducing receipt of SCSs because of their short- and long-term adverse effects (global, 72%), particularly among pulmonologists (72%) and allergists (75%) but less so by otorhinolaryngologists (67%). The use of surgery as a treatment option once pharmacologic therapies fail reached consensus (global, 72%) among pulmonologists (74%) but less among allergists (68%) and otorhinolaryngologists (62%).

### Biologic treatments

Regarding the introduction of biologic treatments (Table E1), panelists reached a consensus on their usefulness in simultaneously managing asthma and CRSwNP symptoms (87%). However, there was a divergence in opinion when it came to prescribing biologics as a first-line therapy. On the one hand, pulmonologists and allergists advocated for considering biologics when patients with SEA and CRSwNP have severe uncontrolled symptoms, ideally as a first-line therapy before resorting to surgical intervention (pulmonologists, 74%; allergists, 100%). On the other hand, otorhinolaryngologists did not reach a consensus on prescribing biologics as first-line therapy and did not favor prioritizing pharmacologic strategies over surgical ones (otorhinolaryngologists, 38%).

### Assessing treatment effectiveness

The panelists reached a consensus that the effectiveness of a treatment for patients with SEA and CRSwNP, as well as the patient’s adherence to therapy, should be evaluated at each follow-up visit. This evaluation should include measuring the recurrence of exacerbations (88%), the need for increased SCS therapy (86%), NP size (73%), and quality of life (76%). However, there was no consensus on how often these follow-up visits should occur (Table E1).

**Fig 3:**
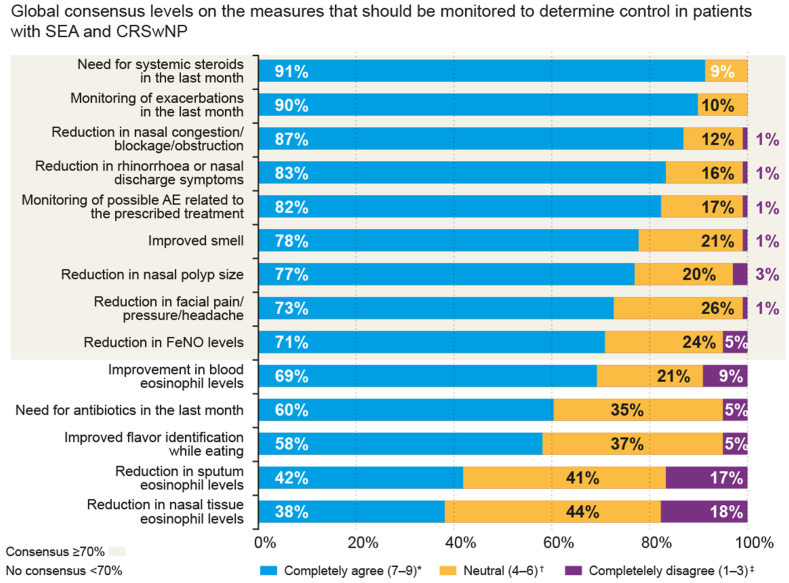
Level of global consensus (all specialties; pulmonologists, allergists, and otorhinolaryngologists) on measures that should be monitored to determine control in patients with SEA and CRSwNP. ∗Completely agree was defined as Likert score of 7-9; †neutral, 4-6, and ‡completely disagree, 1-3.

At follow-up, there was consensus on monitoring various measures or symptoms to determine control, including assessing the need for SCS therapy (91%), the number of exacerbations (90%), nasal congestion (87%), rhinorrhea/obstruction (83%), adverse events (82%), sense of smell (78%), NP size (77%), facial pain/headache (73%), and Feno levels (71%) (Fig 3). It was noted that pulmonologists (72%) and allergists (75%) agreed that blood eosinophil levels were also closely related to the prognosis and severity of inflammation, unlike otorhinolaryngologists, who did not reach a consensus on this statement (43%).

Like the initial assessment consensus outcomes, there was high variability in whether symptom tests or PROs were used to direct the follow-up of comorbid patients (Table E1), with only the ACT test achieving consensus among all specialties (74%). Allergists also reached agreement on the use of the Asthma Control Questionnaire 5 (71%). For the follow-up of patients with CRSwNP, the SNOT-22 test was highlighted as important by allergists (79%) and most of the otorhinolaryngologists (67%).

### Multidisciplinary management and referral

The panelists agreed that the responsibility for effective treatment decision-making and follow-up procedures for patients with SEA and CRSwNP should be shared between pulmonologists/allergists and otorhinolaryngologists (pulmonologists/allergists, 82%; otorhinolaryngologists, 86%).

In their clinical practice, pulmonologists and allergists referred over half (range, 53-63%) of their patients with SEA and CRSwNP to an otorhinolaryngologist. This referral was primarily driven by the need for additional tests and a precise diagnosis to determine the need for surgical intervention for CRSwNP (pulmonologists, 85%; allergists, 89%). The otorhinolaryngologists referred less than half (39%) of their patients with CRSwNP to a pulmonologist/allergist.

There was consensus on the need for a multidisciplinary approach at all stages of disease management (Table E1), from diagnosis (82%) to treatment (83%) and follow-up (79%). The panelists also agreed that specific multidisciplinary units should be established as needed (79%) and that every patient with SEA and CRSwNP should be seen by an otorhinolaryngologist at least once per year (80%).

## Discussion

The OverSEA Delphi study is the largest European initiative providing insights into current clinical practices and recommendations for improving the management of patients with SEA and CRSwNP. This study aimed to elicit broader expert recommendations across a large group of specialists from various European countries to facilitate harmonization in the management and further reinforce any unmet treatment needs of patients with SEA and CRSwNP by using a structured and standardized Delphi survey.

During the initial assessment of CRSwNP in patients with SEA, panelist experts agreed that nasal endoscopy and NP grading should be performed. In addition, pulmonologists and allergists also recommended complementing endoscopy with radiologic/imaging techniques, but otorhinolaryngologists did not. This discrepancy of using imaging techniques may arise because otorhinolaryngologists primarily use endoscopy to evaluate CRSwNP and reserve radiologic/imaging techniques for assessing potential tumors or cancerous growths, clarifying uncertain diagnoses, preparing for surgery, or dealing with complications of chronic rhinosinusitis.^9^ Although there was consensus on using the NP grading system during the initial assessment, the Scientific Committee noted that this system is not universally adopted in all otorhinolaryngologists’ clinical practice, so it may not be familiar to some of them.

Biomarkers play an increasing role in the diagnosis and phenotyping of many conditions, and although there was agreement on the importance of blood eosinophil levels (all specialties) and Feno (pulmonologists and allergists only) as a biomarker in the initial assessment, this was not the case for nasal tissue eosinophil levels or sputum eosinophil counts. Overall, 88% of panelist experts agreed that the assessment of blood eosinophils was important in diagnosing SEA, which aligns with the current treatment guidelines for diagnosing SEA with type 2 inflammatory markers.^25^ The variation in responses regarding the use of blood eosinophils as a diagnostic tool may reflect differences in familiarity with this approach among experts. Furthermore, the lack of consensus on whether to use nasal tissue eosinophils or sputum eosinophil counts is surprising, as tissue eosinophilia is a hallmark of severity in CRSwNP and associated with greater rates of NP recurrence after endoscopic sinus surgery.^26^, ^27^, ^28^ The lack of universal agreement on the value of nasal tissue eosinophil levels could suggest that this biomarker has not been fully integrated into routine clinical practice and that potential barriers exist, such as lack of availability or familiarity with the analytic technique or reimbursement challenges, or that the interventional nature of this test has led to its preferential use in research settings rather than clinical practice. Despite this finding, the Scientific Committee underscored the clinical relevance of nasal tissue eosinophil levels, especially during the initial assessment of patients, as well as for patients who have undergone surgery. They recommended that tissues sampled after sinus surgery should always be collected and analyzed by a pathologist to ensure that this important signal for further treatment planning is not missed. Indeed, nasal tissue eosinophil levels may help to identify patients who are at higher risk of disease recurrence after surgery,^28^ and the values are useful in patients whose disease shows suboptimal response to a biological treatment because it could help guide the next therapeutic steps and potentially a switch to another biologic treatment. Hence, standardization and harmonization of the criteria for obtaining tissue samples after sinus surgery and the assessment of eosinophil levels by a pathologist should be encouraged, which could potentially increase the use of this biomarker.

There was a similar lack of consensus for most symptom tests or PROs, with only the ACT achieving global consensus, and consensus among pulmonologists and allergists. Panelists reached consensus on, and hence recognized, the importance of testing the reduction in or loss of smell and evaluating the patient quality of life, but no specific PRO or visual analog scale test emerged as a preferred choice for CRSwNP. These findings reveal a considerable variability in clinical practices, indicate a limited integration of symptom tests/PROs in CRSwNP management, and highlight a significant area for improvement. Therefore, the Steering Committee recommended an increase in the awareness regarding the use of symptoms tests, particularly those that are easy to perform and implement, such as visual analog scale–based PROs that could be completed by patients in the waiting room before consultation.

While there was agreement on the primary treatment objectives (reduce asthma exacerbations and achieve asthma control while simultaneously addressing NP symptoms and reducing NP size), some discrepancies emerged regarding deploying specific therapies, like biologics. While all specialties recognized the usefulness of biologics in simultaneously managing asthma and CRSwNP symptoms, opinion was divided regarding the use of biologics as a first-line therapy in comorbid patients. Indeed, pulmonologists and allergists advocated for biologics as a first-line therapy for severe uncontrolled symptoms before surgical intervention, while otorhinolaryngologists did not reach a consensus on prioritizing pharmacologic strategies over surgical ones. These results indicate that otorhinolaryngologists view surgery as the first-line therapy for NP, potentially because 30-60% of patients experience complete disease control after surgery.^9^ Furthermore, there was a lack of consensus between panelists on how often the efficacy of a therapeutic regimen should be evaluated; this may be attributed to limited guidance in the current treatment guidelines.^13^ These discrepancies in treatment choices and dosing frequency across specialists suggest a need for standardized education on the assessment and management of patients with SEA and CRSwNP among specialists. The Steering Committee highlighted that there are advantages of using targeted treatments for both lower respiratory and ear, nose, and throat symptoms in severe or uncontrolled patients with SEA and CRSwNP, such as reducing the need for SCS therapy or recurrent surgery; however, further research into the duration of receipt and cost-effectiveness of biologics in Europe is required.

The panelists agreed on shared responsibility between pulmonologists/allergists and otorhinolaryngologists for effective treatment decision-making and follow-up procedures for patients with SEA and CRSwNP. That said, while pulmonologists and allergists referred over half of their patients with SEA and CRSwNP to an otorhinolaryngologist, otorhinolaryngologists only referred just over a third of their patients with CRSwNP to a pulmonologist/allergist. These results highlight the need to establish more multidisciplinary teams across hospitals in different European countries, and a potential lack of awareness among specialists about the importance of managing their patients with SEA and CRSwNP with the other relevant specialties. This lack of awareness might originate in an absence of referral procedures or protocols between specialties in some hospitals, or a respective lack of referral systems or use thereof in private care. Ideally, there should be a reciprocal collaboration between pulmonologists, allergists, and otorhinolaryngologists, in partnership with primary care physicians, in the management of patients with SEA and CRSwNP, especially for severe or uncontrolled disease. It is also important to highlight that pulmonologists/allergists primarily consider referral to otorhinolaryngologists when upper respiratory symptoms are prominent or when surgery is needed. Likewise, between-specialist referrals may be important. For example, an allergist could assess the impact of an allergic comorbidity, while a pulmonologist may be useful for full physiological evaluations to identify additional comorbidities. Broader collaboration in patient management earlier in the disease pathway could thus be more beneficial to the patient.

While the outcomes of the OverSEA study are well substantiated because of its design and the large cohort of contributing physicians, some factors require careful consideration when interpreting the Delphi study findings. These include sample heterogeneity, which could lead to variations in clinical practice and perspectives; the sample’s predominant representation of pulmonologists, which may limit the generalizability of the findings to other specialties; differences in health care systems and reimbursement policies across regions or countries, which could contribute to the varied perspectives observed; and local factors that may impact the feasibility and applicability of certain recommendations, such as the assessment of biomarkers. It is also important to note that although the Delphi survey is designed for unbiased responses because of its anonymity, interpretation of the survey questions is highly dependent on responder expertise and level of engagement, and it therefore could lead to variability in outcomes. Additionally, this was a sponsored study, but panelists were not aware of this so their responses would not be influenced. Also, while the management of patients with SEA and CRSwNP involves diverse specialties across European countries, the involvement of allergists may not be consistent across all countries. Finally, some important aspects of asthma and CRSwNP management, such as the concept of clinical remission, were not considered because this Delphi questionnaire was developed before the concept of long-term remission was introduced in the field of asthma and CRSwNP management.

In conclusion, the OverSEA study provides insights into current clinical practices and indicates that patients with SEA should also be assessed for comorbid upper airways diseases, such as CRSwNP. Opinions on the use of multidisciplinary approaches in the management of SEA and CRSwNP have been previously described in qualitative discussions,^29^^,^^30^ but no structured Delphi across-Europe study has been conducted until now. Any patient with CRSwNP should be screened for comorbid asthma. The initial evaluation of a patient with SEA should include current asthma symptom control (via ACT), evaluation of loss of smell, nasal endoscopy, history of AERD/NERD, adherence to inhaled and intranasal corticosteroids, and receipt of SCS therapy. The existence of a pathophysiologic continuum of type 2 inflammation between the lower and upper airways, often referred to as *united airway disease,* suggests potential benefits of a collaborative or multidisciplinary team approach to disease management. Forming multidisciplinary groups could lead to the development of guidelines or checklists, and other resources for national and international implementation. This could encompass the endorsement of common symptom tests and/or PROs for evaluating SEA and CRSwNP, particularly during the initial assessment, during follow-up, and in relation to biologics.

This Delphi survey also highlights areas that require attention, including potential referral challenges, defining a biomarker matrix for screening, benefits and potential risks of surgical interventions, and encouraging timely intervention with biologic therapy in patients with SEA and comorbid CRSwNP.

## Disclosure statement

This study was supported by GSK, which funded Adelphi Targis to design and conduct the survey following Delphi methodology and to provide medical writing support to the authors. The authors are solely responsible for opinions, conclusions, and interpreting results, and they approved the final content. The authors took the decision to submit for publication, and the sponsor did not restrict access to the data or the statements made.

Disclosure of potential conflict of interest: C. Bachert has attended advisory boards/received lecture fees from GSK, Novartis, 10.13039/100004339Sanofi and 10.13039/100009857Regeneron, and Insmed. G. Brusselle has attended advisory boards/received lecture fees from AstraZeneca, Chiesi, GSK, Novartis, and Sanofi and Regeneron; and attended advisory boards from Boehringer-Ingelheim and MSD. J. A. Castillo Vizuete has received payment/honoraria from AstraZeneca, GSK, and ALK-Abelló; has received support for attending meetings/travel from AstraZeneca, GSK, and Sanofi-Genzyme; has participated on data safety monitoring board/advisory board of AstraZeneca, GSK, and ALK-Abelló; and is chairman of the Rhinitis, Rhinosinusitis and Nasal Polyposis Group in Asthma Section Sociedad Española de Neumologia (SEPAR). I. Dávila has received grants/contracts from 10.13039/501100004587Instituto de Salud Carlos III, Junta de Castilla y León, and Thermo Fisher Scientific; consulting fees from Allergy Therapeutics, AstraZeneca, GSK, MSD, Novartis, and Sanofi; and payments/honoraria for lectures from Allergy Therapeutics, Sanofi, AstraZeneca, MSD, GSK, Chiesi, Novartis, and Diater. M. Laudien has received support from GSK; grants/contracts from Olympus Deutschland GmbH & Europa SE & Co KG, Brainlab Sales, AEDA, Flagon, and Medtronic; payments/honoraria for lectures from Novartis Pharma, Sanofi-Aventis Deutschland, GSK, and CSL Vifor; and has played a leadership/fiduciary role in AG Rhinologie/Rhinochirurgie DGHNO, John Grube Foundation, and CRS Register. V. Seccia has participated in advisory boards, received payments/honoraria for lectures, and participated on data safety monitoring board/advisory board from GSK, Sanofi, and AstraZeneca; and has received support for attending meetings/travel from Sanofi. P. Schmid-Grendelmeier has attended advisory boards, contributed to the discussion, and received honoria from AstraZeneca, GSK, Novartis, and Sanofi. A. Vultaggio has attended advisory boards and received payments/honoraria for lectures from AstraZeneca, GSK, Sanofi, and Novartis. K. Kallinikou and L. Walrave are employees of GSK and hold stocks/shares in GSK. L. Klimek has received grants/contracts from Allergopharma, Viatris, LETI Pharma, and Stallergenes; grants from HAL Allergie, ALK-Abelló, Quintiles, Lofarma, Allergopharma, Viatris, HAL Allergie, ALK-Abelló, LETI Pharma, Stallergenes, Quintiles, Sanofi, Lofarma, Allergy Therapeutics, AstraZeneca, GSK, Inmunotek, Cassella med, Novartis, Regeneron Pharmaceuticals, and ROXALL Medizin; consulting fees from Allergopharma, GSK, Viatris, LETI Pharma, Novartis, Stallergenes, Sanofi, and AstraZeneca; and has other financial or nonfinancial interests related to: president of German Allergy Society AeDA, vice president of German Academy for Allergy and Clinical Immunology and the European Academy of Allergy and Clinical Immunology, and member of DGHNO, HNO-BV, and GPA.
