# Supplementary material for: Consensus from European experts on severe eosinophilic asthma and chronic rhinosinusitis with nasal polyps: Results from the OverSEA Delphi study
Source: J Allergy Clin Immunol Glob. 2025 Jul 3;4(4):100529. doi: 10.1016/j.jacig.2025.100529 (PMC12359228; doi:10.1016/j.jacig.2025.100529)
Supplement: Supplementary Figs and Tables [file mmc1.docx]

Online Repository materials
Figure E1**.** Overview of Delphi process employed to achieve consensus recommendations on the management of patients with Severe Asthma with an Eosinophilic Phenotype (SEA) and Chronic Rhinosinusitis with Nasal Polyps (CRSwNP).

**
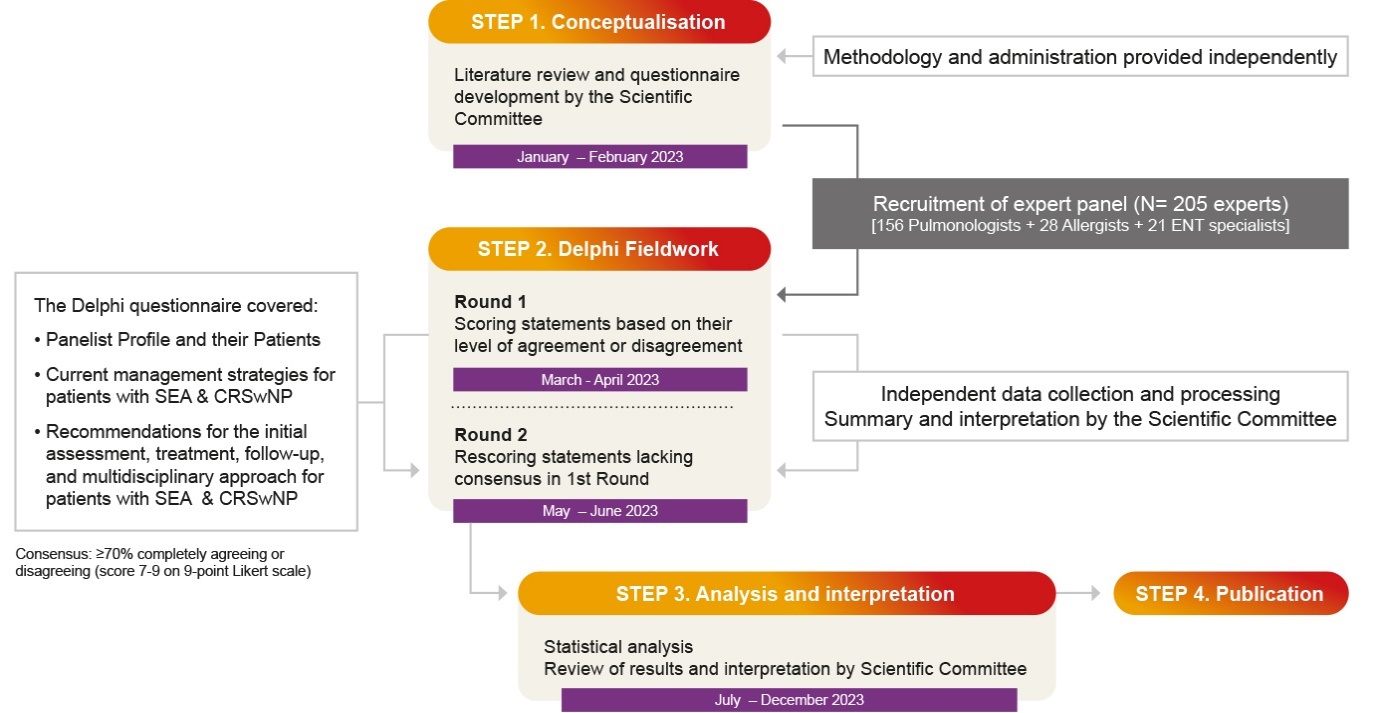
**

Table E1**.** Delphi Questionnaire and Global Consensus Results (all specialties; pulmonologists, allergists, and otorhinolaryngologists).

| **Question** | **Statement no.** | **Statement** | **% Completely disagree**  **(1-3)** | **% Neutral (4-6)** | **% Completely agree (7-9)** | **Consensus decision** |
| --- | --- | --- | --- | --- | --- | --- |
| **According to YOUR OPINION, please state how far you agree with the statements presented below related to the upper respiratory tract comorbidities that should be considered for the proper management of patients with SEA** | **S1** | **Chronic rhinosinusitis with nasal polyps (CRSwNP)** | **0.0** | **11.7** | **88.3** | **Consensus** |
|  | **S2** | **Allergic rhinitis** | **1.5** | **19.5** | **79.0** | **Consensus** |
|  | **S3** | **Chronic rhinosinusitis without nasal polyps (CRSsNP)** | **2.9** | **20.5** | **76.6** | **Consensus** |
|  | **S4** | **Aspirin/non-steroidal anti-inflammatory-exacerbated respiratory disease (AERD/NERD)** | **2.9** | **26.3** | **70.7** | **Consensus** |
|  | S5 | Acute rhinosinusitis | 7.3 | 36.6 | 56.1 | No consensus |
|  | S6 | Obstructive sleep apnea (OSA) | 5.4 | 40.5 | 54.1 | No consensus |
| **According to YOUR OPINION, please state how far you agree with the statements presented below related to the importance of the assessment of upper respiratory tract comorbidities in adult patients with SEA** | **S7** | **Early comprehensive management of SEA and its associated comorbidities would reduce the use of oral steroids. avoiding their adverse effects.** | **0.0** | **9.8** | **90.2** | **Consensus** |
|  | **S8** | **Proper management of comorbidities would improve patients' quality of life** | **0.5** | **9.8** | **89.8** | **Consensus** |
|  | **S9** | **The early detection of comorbidities associated with SEA would facilitate the proper therapeutic management of the patients** | **0.0** | **12.7** | **87.3** | **Consensus** |
|  | **S10** | **Proper management of comorbidities would contribute to good SEA control** | **0.5** | **12.7** | **86.8** | **Consensus** |
|  | **S11** | **The routine detection of comorbidities associated with SEA would make it possible to refer patients to the right specialists for early management** | **0.5** | **16.1** | **83.4** | **Consensus** |
| **According to YOUR OPINION, please state how far you agree with the items presented below related to the aspects that should be evaluated during the initial assessment and diagnosis of CRSwNP in adult patients with SEA** | **S12** | **Lack of asthma control** | **1.5** | **13.2** | **85.4** | **Consensus** |
|  | **S13** | **Increase in the need for oral steroid boosts/per year** | **1.5** | **15.6** | **82.9** | **Consensus** |
|  | **S14** | **Use of nasal corticosteroids** | **2.0** | **21.0** | **77.1** | **Consensus** |
|  | **S15** | **Presence of aspirin/non-steroidal anti-inflammatory-exacerbated respiratory disease (AERD/NERD)** | **1.5** | **22.0** | **76.6** | **Consensus** |
|  | S16 | Late-onset asthma | 3.4 | 29.8 | 66.8 | No consensus |
|  | S17 | Patient family history | 2.4 | 42.4 | 55.1 | No consensus |
|  | S18 | Increase in nasal symptoms when eating certain foods (e.g., cheese or alcohol) | 6.3 | 47.3 | 46.3 | No consensus |
| **According to YOUR OPINION, please state how far you agree with the items presented below related to the clinical symptoms that should be evaluated during the initial assessment and diagnosis of CRSwNP in adult patients with SEA** | **S19** | **Reduction in or loss of smell** | **0.0** | **9.8** | **90.2** | **Consensus** |
|  | **S20** | **Nasal congestion / blockage / obstruction** | **0.5** | **9.8** | **89.8** | **Consensus** |
|  | **S21** | **Rhinorrhea or nasal discharge (anterior/posterior nasal drip)** | **1.0** | **13.7** | **85.4** | **Consensus** |
|  | **S22** | **Facial pain/pressure/headache** | **2.0** | **20.0** | **78.0** | **Consensus** |
|  | **S23** | **Reduction in or loss of flavor identification while eating** | **0.5** | **22.9** | **76.6** | **Consensus** |
|  | S24 | Sleep disorders | 4.4 | 28.3 | 67.3 | No consensus |
|  | S25 | Hearing problems | 9.3 | 45.4 | 45.4 | No consensus |
| **According to YOUR OPINION, please state how far you agree with the items presented below related to the biomarkers that should be evaluated during the initial assessment and diagnosis of CRSwNP in adult patients with SEA** | **S26** | **Blood eosinophil levels** | **0.5** | **11.7** | **87.8** | **Consensus** |
|  | **S27** | **Fractional exhaled nitric oxide (FeNO)** | **1.5** | **20.5** | **78.0** | **Consensus** |
|  | S28 | Nasal tissue eosinophil levels | 9.3 | 37.6 | 53.2 | No consensus |
|  | S29 | Sputum eosinophil count | 10.7 | 36.6 | 52.7 | No consensus |
| **According to YOUR OPINION, please state how far you agree with the items presented below related to the tools or clinical tests that should be evaluated during the initial assessment and diagnosis of CRSwNP in adult patients with SEA** | **S30** | **Nasal endoscopy** | **2.9** | **12.7** | **84.4** | **Consensus** |
|  | **S31** | **Nasal polyp endoscopic grading system** | **4.9** | **21.5** | **73.7** | **Consensus** |
|  | **S32** | **Radiologic evaluation and imaging studies (e.g., computerized tomography (CT), cone-beam CT, and magnetic resonance imaging (MRI))** | **2.4** | **24.9** | **72.7** | **Consensus** |
|  | S33 | Smell test (e.g., UPSIT or European Sniffin’ Sticks) | 10.7 | 50.2 | 39.0 | No consensus |
|  | S34 | Nasal airflow assessment (Peak nasal inspiratory flow (PNIF) and/or Rhinomanometry) | 6.8 | 55.1 | 38.0 | No consensus |
|  | S35 | Nasal cytology | 12.7 | 50.7 | 36.6 | No consensus |
| **According to YOUR OPINION, please state how far you agree with the items presented below related to the symptom tests/PROs that should be evaluated during the initial assessment and diagnosis of CRSwNP in adult patients with SEA** | **S36** | **Asthma Control Test (ACT)** | **3.4** | **21.0** | **75.6** | **Consensus** |
|  | S37 | Visual Analogue Scale (VAS) for CRSwNP overall symptoms | 2.4 | 34.1 | 63.4 | No consensus |
|  | S38 | Asthma Quality of Life Questionnaire (AQLQ) | 3.4 | 34.6 | 62.0 | No consensus |
|  | S39 | Asthma Control Questionnaire 5 (ACQ-5) | 3.4 | 35.1 | 61.5 | No consensus |
|  | S40 | Sino-nasal Outcome Test (SNOT-22) | 2.9 | 36.1 | 61.0 | No consensus |
|  | S41 | VAS for loss of smell | 3.9 | 36.1 | 60.0 | No consensus |
|  | S42 | Asthma Control Questionnaire 6 (ACQ-6) | 2.9 | 37.6 | 59.5 | No consensus |
|  | S43 | VAS for nasal obstruction | 3.4 | 38.0 | 58.5 | No consensus |
|  | S44 | VAS for rhinorrhea | 3.4 | 45.9 | 50.7 | No consensus |
|  | S45 | Total nasal symptom score RSOM-31 | 7.8 | 48.8 | 43.4 | No consensus |
|  | S46 | VAS for facial pain | 8.8 | 50.2 | 41.0 | No consensus |
|  | S47 | Smell test (e.g. UPSIT or European Sniffin’ Sticks) | 10.7 | 50.2 | 39.0 | No consensus |
|  | S48 | St. George’s Respiratory Questionnaire (SGRQ) | 18.0 | 44.4 | 37.6 | No consensus |
| **According to YOUR OPINION, please state how far you agree with the statements presented below related to the procedures that should be followed in the initial assessment and diagnosis of CRSwNP in adult patients with SEA** | **S49** | **When malignancy is suspected, a histological examination must be performed** | **0.5** | **9.3** | **90.2** | **Consensus** |
|  | **S50** | **The patient's health history and concomitant base conditions should be taken into account when making the differential diagnosis** | **0.5** | **22.4** | **77.1** | **Consensus** |
|  | **S51** | **A nasal endoscopy should always be performed to evaluate NP size and severity** | **2.0** | **22.4** | **75.6** | **Consensus** |
|  | S52 | Endoscopic confirmation is sufficient without other symptoms for diagnosis NP | 5.9 | 34.6 | 59.5 | No consensus |
|  | S53 | Loss of smell should be evaluated by means of an objective test and/or VAS smell evaluation as part of the NP differential diagnosis procedure | 4.9 | 38.0 | 57.1 | No consensus |
|  | S54 | CT scanning studies should be performed early in the disease course and not after first-line therapy failure | 8.8 | 35.1 | 56.1 | No consensus |
|  | S55 | The initial assessment of SEA should always include a nasal QoL assessment though validated questionnaires (SNOT-22 or VAS scales) | 2.4 | 42.4 | 55.1 | No consensus |
|  | S56 | Multi-detector CT (MDCT) scanners and cone beam CT studies should be prioritized over traditional CT | 5.4 | 46.8 | 47.8 | No consensus |
|  | S57 | Performing an adequate histopathological evaluation is essential in establishing the degree of sinonasal inflammation | 9.3 | 45.4 | 45.4 | No consensus |
| **According to YOUR OPINION, please state how far you agree with the statements presented below related to the actions to be performed following the diagnosis of nasal polyps in patients with SEA** | **S58** | **A patient with SEA and nasal polyps should be referred to an ENT specialist* to assess whether surgery is required** | **1.0** | **12.2** | **86.8** | **Consensus** |
|  | S59 | A patient with SEA and CRSwNP should be referred to an ENT specialist* who will decide upon the pharmacological treatment | 7.8 | 30.7 | 61.5 | No consensus |
| **According to YOUR OPINION, please state how far you agree with the statements presented below related to the treatment objectives that should be considered in the treatment of patients with SEA and CRSwNP** | **S60** | **The treatment objective should be to reduce the asthma exacerbations caused partly by the presence of NP** | **0.0** | **9.8** | **90.2** | **Consensus** |
|  | **S62** | **The treatment objective should consist of selecting the most suitable treatment to control asthma and NP at the same time** | **0.5** | **10.2** | **89.3** | **Consensus** |
|  | **S63** | **The treatment objective should be to reduce the NP symptoms that have a substantial effect on patients' quality of life** | **0.5** | **11.2** | **88.3** | **Consensus** |
|  | **S64** | **The treatment objective should be to reduce the use of oral steroids** | **0.0** | **12.2** | **87.8** | **Consensus** |
|  | **S65** | **The treatment objective should be to improve the NP symptoms that have a substantial effect on asthma control** | **0.0** | **13.7** | **86.3** | **Consensus** |
|  | **S66** | **The use of biological drugs as first-line therapy should be promoted in severe uncontrolled patients with SEA and CRSwNP since they make it possible to treat the asthma and the polyps simultaneously** | **1.5** | **14.6** | **83.9** | **Consensus** |
|  | **S67** | **The treatment objective should be to focus on reducing nasal polyp size in order to improve asthma control** | **2.9** | **18.0** | **79.0** | **Consensus** |
|  | **S68** | **The treatment objective should be to achieve and maintain clinical control of SEA with minimal use of medication despite the comorbidities** | **1.0** | **21.0** | **78.0** | **Consensus** |
|  | **S69** | **The treatment of SEA and NP should prioritize pharmacological strategies** | **1.5** | **24.4** | **74.1** | **Consensus** |
|  | **S70** | **Surgery should be used as a last resort in severe uncontrolled patients who do not respond to pharmacological treatment** | **4.4** | **24.9** | **70.7** | **Consensus** |
| **According to YOUR OPINION, please state how far you agree with the statements presented below related to the treatment decisions that should be taken for patients with SEA and CRSwNP** | **S71** | **In the event of a suspected worsening of the NP, pharmaceutical treatment should be reviewed** | **0.5** | **11.7** | **87.8** | **Consensus** |
|  | **S72** | **In SEA patients with CRSwNP, the frequency of a recurring need for systemic steroids should be considered as poor disease control** | **0.5** | **16.1** | **83.4** | **Consensus** |
|  | **S73** | **There is a need for novel therapies in patients with uncontrolled CRSwNP after surgery or after the failure of other established therapies** | **2.0** | **17.1** | **81.0** | **Consensus** |
|  | **S74** | **Surgery should be the treatment option for CRSwNP patients when medical treatment fails** | **2.9** | **24.9** | **72.2** | **Consensus** |
|  | **S75** | **The use of systemic treatment should be reduced due to their short and long-term adverse effects** | **3.9** | **23.9** | **72.2** | **Consensus** |
| **According to YOUR OPINION, please state how far you agree with the statements presented below related to the considerations to be taken into account when introducing a biologic for the treatment of patients with SEA and CRSwNP** | **S76** | **A biologic that reduces clinically significant exacerbations in patients with CRSwNP and SEA should be chosen** | **0.5** | **12.2** | **87.3** | **Consensus** |
|  | **S77** | **A biologic that directly impacts the pathogenesis of CRSwNP and reduces type 2 inflammation should be selected** | **1.0** | **14.1** | **84.9** | **Consensus** |
|  | **S78** | **A biologic that directly targets specific interleukins that impact the recurrence of SEA and CRSwNP should be chosen** | **2.0** | **19.0** | **79.0** | **Consensus** |
|  | **S79** | **A biologic whose mechanism of action impacts the recruitment and survival of eosinophils in tissues should be chosen** | **0.5** | **25.4** | **74.1** | **Consensus** |
|  | **S80** | **A biological treatment should be the first-line therapy for severe, uncontrolled patients with SEA and CRSwNP** | **6.3** | **19.5** | **74.1** | **Consensus** |
|  | **S81** | **A biologic that delivers a sustained reduction in blood eosinophil count over time should be chosen** | **4.9** | **23.9** | **71.2** | **Consensus** |
| **According to YOUR OPINION, please state how far you agree with the statements presented below related to the criteria for the evaluation of the efficacy of the treatment for SEA and CRSwNP** | **S82** | **One of the main criteria for determining a treatment's efficacy should be the asthma control** | **0.0** | **10.7** | **89.3** | **Consensus** |
|  | **S83** | **The reduction in the number of exacerbations is indispensable for a treatment to be considered efficacious** | **0.5** | **11.2** | **88.3** | **Consensus** |
|  | **S84** | **The reduction in the need for systemic steroids indicates therapeutic efficacy** | **0.0** | **13.7** | **86.3** | **Consensus** |
|  | **S85** | **Improved scores in quality-of-life questionnaires indicate that a treatment is proving to be effective** | **1.5** | **22.9** | **75.6** | **Consensus** |
|  | **S86** | **One of the main criteria for determining a treatment's efficacy should be the reduction in polyp size** | **3.9** | **23.4** | **72.7** | **Consensus** |
|  | S87 | In general terms, the efficacy of the prescribed therapeutic regimen should be evaluated every 3 months | 4.4 | 30.2 | 65.4 | No consensus |
|  | S88 | Improved smell is an indispensable parameter for evaluating the therapeutic regimens efficacy | 3.4 | 32.2 | 64.4 | No consensus |
|  | S89 | In general terms, the efficacy of the prescribed therapeutic regimen should be evaluated every 6 months | 3.4 | 34.6 | 62.0 | No consensus |
|  | S90 | In general terms, the efficacy of the prescribed therapeutic regimen should be evaluated at least once a year | 21.0 | 33.7 | 45.4 | No consensus |
|  | S91 | In general terms, the efficacy of the prescribed therapeutic regimen should be evaluated every 9 months | 26.3 | 44.9 | 28.8 | No consensus |
| **According to YOUR OPINION, please state how far you agree with the statements presented below related to the aspects that should be controlled/monitored during the follow-up of patients with SEA and CRSwNP** | **S92** | **Need for systemic steroids in the last month** | **0.0** | **8.8** | **91.2** | **Consensus** |
|  | **S93** | **Monitoring of exacerbations in the last month** | **0.5** | **9.8** | **89.8** | **Consensus** |
|  | **S94** | **Reduction in nasal congestion/blockage /obstruction** | **1.0** | **12.2** | **86.8** | **Consensus** |
|  | **S95** | **Reduction in rhinorrhea or nasal discharge symptoms** | **1.0** | **15.6** | **83.4** | **Consensus** |
|  | **S96** | **Monitoring of possible adverse events (AE) related to the prescribed treatment** | **1.0** | **16.6** | **82.4** | **Consensus** |
|  | **S97** | **Improved smell** | **1.5** | **21.0** | **77.6** | **Consensus** |
|  | **S98** | **Reduction in nasal polyp size** | **2.9** | **20.0** | **77.1** | **Consensus** |
|  | **S99** | **Reduction in facial pain/pressure/headache** | **1.5** | **25.9** | **72.7** | **Consensus** |
|  | **S100** | **Reduction in FeNO levels** | **5.4** | **23.9** | **70.7** | **Consensus** |
|  | S101 | Improvement in blood eosinophil levels | 9.3 | 21.5 | 69.3 | No consensus |
|  | S102 | Need for antibiotics in the last month | 4.9 | 34.6 | 60.5 | No consensus |
|  | S103 | Improved flavor identification while eating | 4.9 | 37.1 | 58.0 | No consensus |
|  | S104 | Reduction in sputum | 16.6 | 41.5 | 42.0 | No consensus |
|  | S105 | Reduction in nasal tissue eosinophil levels | 17.6 | 44.4 | 38.0 | No consensus |
| **According to YOUR OPINION, please state how far you agree with the items presented below related to the clinical tests that should be used in the follow-up of patients with CRSwNP and SEA** | **S106** | **Nasal endoscopy** | **3.9** | **18.5** | **77.6** | **Consensus** |
|  | S107 | Nasal polyp endoscopic grading system | 3.4 | 35.1 | 61.5 | No consensus |
|  | S108 | Radiologic evaluation and imaging studies (e.g., computerized tomography (CT). cone-beam CT. and magnetic resonance imaging (MRI)) | 11.7 | 41.0 | 47.3 | No consensus |
|  | S109 | Nasal airflow assessment (Peak nasal inspiratory flow (PNIF) and/or Rhinomanometry) | 9.3 | 47.3 | 43.4 | No consensus |
|  | S110 | Smell test (e.g., UPSIT or European Sniffin’ Sticks) | 8.3 | 53.7 | 38.0 | No consensus |
|  | S111 | Nasal cytology | 22.9 | 46.3 | 30.7 | No consensus |
| **According to YOUR OPINION, please state how far you agree with the items presented below related to the PROs that should be used in the follow-up of patients with CRSwNP and SEA** | **S112** | **Asthma Control Test (ACT)** | **3.9** | **22.0** | **74.1** | **Consensus** |
|  | S113 | Asthma Control Questionnaire 6 (ACQ-6) | 7.3 | 33.7 | 59.0 | No consensus |
|  | S114 | Asthma Quality of Life Questionnaire (AQLQ) | 5.9 | 35.1 | 59.0 | No consensus |
|  | S115 | Visual Analogue Scale (VAS) for nasal obstruction | 2.4 | 39.0 | 58.5 | No consensus |
|  | S116 | Asthma Control Questionnaire 5 (ACQ-5) | 5.4 | 36.1 | 58.5 | No consensus |
|  | S117 | VAS for CRSwNP overall symptoms | 2.9 | 39.5 | 57.6 | No consensus |
|  | S118 | VAS for loss of smell | 3.4 | 39.5 | 57.1 | No consensus |
|  | S119 | Sino-nasal Outcome Test (SNOT-22) | 3.9 | 39.0 | 57.1 | No consensus |
|  | S120 | VAS for rhinorrhea | 5.4 | 47.3 | 47.3 | No consensus |
|  | S121 | St. George’s Respiratory Questionnaire (SGRQ) | 17.1 | 42.4 | 40.5 | No consensus |
|  | S122 | Smell test (e.g., UPSIT or European Sniffin’ Sticks) | 8.3 | 53.7 | 38.0 | No consensus |
|  | S123 | Total nasal symptom score RSOM-31 | 10.2 | 52.2 | 37.6 | No consensus |
|  | S124 | VAS for facial pain | 10.2 | 58.0 | 31.7 | No consensus |
| **According to YOUR OPINION, please state how far you agree with the items presented below related to the procedures that should be applied in the follow-up of patients with CRSwNP and SEA** | **S125** | **The responsibility for following up a patient with SEA and NP should be shared by a pulmonologist/allergist and an ENT specialist*** | **1.0** | **16.6** | **82.4** | **Consensus** |
|  | S126 | The pulmonologist/allergist should be mainly in charge of following up patients with SEA and NP | 6.3 | 29.8 | 63.9 | No consensus |
|  | S127 | In general terms, NP size should be endoscopically reviewed every 6 months | 6.8 | 35.6 | 57.6 | No consensus |
|  | S128 | In general terms, the NP symptoms should be reviewed every 3 months | 10.7 | 42.9 | 46.3 | No consensus |
|  | S129 | In general terms, quality of life should be reviewed every 9 months | 16.1 | 46.3 | 37.6 | No consensus |
| **According to YOUR OPINION, please state how far you agree with the statements presented below related to the actions that should be performed in the event of worsening during the follow-up and management of CRSwNP in adult patients with SEA** | **S130** | **Treatment should be re-evaluated and changed if necessary** | **0.0** | **10.7** | **89.3** | **Consensus** |
|  | **S131** | **The patients' degree of treatment adherence should be evaluated** | **1.0** | **10.7** | **88.3** | **Consensus** |
|  | **S132** | **The NP symptoms should be re-evaluated** | **0.5** | **12.7** | **86.8** | **Consensus** |
|  | **S133** | **NP size should be re-evaluated in order to appraise the need for surgery** | **2.0** | **12.7** | **85.4** | **Consensus** |
|  | **S134** | **The patient should be referred to the ENT specialist* for a re-evaluation of the diagnosis and treatment** | **1.0** | **14.1** | **84.9** | **Consensus** |
| **According to YOUR OPINION, please state how far you agree with the statements below related to the aspects related to multidisciplinary management of CRSwNP in adult patients with SEA** | **S135** | **The treatment of NP in a patient with SEA should be decided by a multidisciplinary team consisting of a respiratory specialist (pulmonologist and/or allergist) and an ENT specialist*** | **2.4** | **14.6** | **82.9** | **Consensus** |
|  | **S136** | **The diagnosis of NP in a patient with SEA should be performed by a multidisciplinary team consisting of a respiratory specialist (pulmonologist and/or allergist) and an ENT specialist*** | **2.4** | **15.6** | **82.0** | **Consensus** |
|  | **S137** | **Every patient with SEA and CRSwNP should be seen by an ENT specialist* at least once a year** | **1.5** | **18.5** | **80.0** | **Consensus** |
|  | **S138** | **Multidisciplinary management units should be created for the treatment of patients with SEA and CRSwNP** | **2.0** | **19.5** | **78.5** | **Consensus** |
|  | **S139** | **The follow-up of NP in a patient with SEA should be performed by a multidisciplinary team consisting of a respiratory specialist (pulmonologist and/or allergist) and an ENT specialist*** | **2.9** | **18.5** | **78.5** | **Consensus** |
|  | S140 | Patients with SEA and CRSwNP should only be referred to the ENT specialist* if they do not improve with pharmacological treatment | 32.2 | 32.7 | 35.1 | No consensus |
|  | S141 | Patients with SEA and CRSwNP should only be referred to the ENT specialist* if they need surgery | 42.4 | 29.3 | 28.3 | No consensus |

*Referred to as otorhinolaryngologist within the text.
